# Mental Health Support Programme BOJE (Colours): Two Years of Supporting LGBTQIA+ Mental Health in Croatia

**DOI:** 10.1192/j.eurpsy.2026.10759

**Published:** 2026-07-31

**Authors:** A. Vuk, J. Hubler, I. Barun, L. Mijalkovic, M. Supe, J. Pravdic, V. V. Dogas, V. Grosic

**Affiliations:** 1 Department of integrativne psychiatry; 2 Addiction Day Hospital; 3 Eating Disorders Day Hospital; 4 Department of Psychotherapeutic Treatment, University Psychiatric Hospital Sveti Ivan, Zagreb, Croatia

## Abstract

**Introduction:**

Minority stress remains a central factor in the mental health of sexual and gender minorities often resulting in elevated distress, anxiety, depression, suicidality, and substance misuse (Moorhead, B., Lewis, H.K. & Arnull, L. LGBT sexuality and gender minority experiences of minority stress: a comparison of models and theories. Qual Quant 58, 3973–4001 (2024)). Building on insights from previous research, the Mental Health Support Programme BOJE (Colours) was launched to offer comprehensive support tailored to the needs of LGBTQIA+ individuals within the Croatian public mental healthcare system.

**Objectives:**

The aim is to present our two year experinece with the BOJE - Mental Health Support Programme that aims to address minority stress to offer specialized support tailored to the unique needs of LGBTIQ+ individuals, fostering resilience and well-being.

**Methods:**

In October 2023, a multidisciplinary team at the University Psychiatric Hospital Sveti Ivan in Zagreb, Croatia, formed a Mental Health Protection Programme BOJE which consists of a counseling center, an outpatient clinic and a three-month therapeutic and educational cycle for LGBTQIA+ users. Over time, the programme has expanded to include support group, psychotherapeutic with families and a platform was provided for training healthcare professionals in LGBTQIA+ affirmative practice.

**Results:**

To date, we have carried out more than 1,200 interventions — including counseling, psychiatric consultations, reviews, and psychotherapy. Four cycles of closed-group workshops have been completed with a very low dropout rate, and a fifth cycle is currently underway. The support group now brings together participants from all previous cycles. n the evaluation of the work so far, we see that the input data show a high level of distress, while the preliminary output data shows improvement. Across both group formats, we observed rapid development of cohesion, stronger interpersonal support, greater participation, improved outcomes, and consistently low dropout rates.

**Conclusions:**

The BOJE programme represents the first structured mental health intervention for LGBTQIA+ people within the Croatian healthcare system. It shows that affirmative, structured interventions can effectively address the impact of minority stress and improve the mental health of LGBTQIA+ people. Our experience highlights the rapid development of group cohesion, the protective role of family acceptance, and the value of safe therapeutic spaces for identity exploration and resilience. Together, these outcomes demonstrate that inclusive mental health care not only fosters wellbeing but also strengthens belonging, community support, and hope.

**Disclosure of Interest:**

None Declared

